# Establishment and efficacy validation of sex-age-specific diagnostic cutoffs for sarcopenia: targeting the decline inflection points of muscle health indicators in community-dwelling middle-aged and elderly

**DOI:** 10.3389/fragi.2026.1792871

**Published:** 2026-06-25

**Authors:** Wenwen Zhao, Mingjie Meng, Nuoya Li, Yiheng Geng, Hangtian Yu, Jinjia Zhang, Min Zhang, Meijing Song, Rongying Wang

**Affiliations:** 1 General Practice Department, The Second Hospital of Hebei Medical University, Shijiazhuang, Hebei, China; 2 Department of Cardiology, Division III, The Second Hospital of Hebei Medical University, Shijiazhuang, Hebei, China; 3 College of Public Health, Hebei Medical University, Shijiazhuang, Hebei, China; 4 Community Service Centre of Lianmeng Subdistrict Office, Xinhua District, Shijiazhuang, Hebei, China

**Keywords:** sarcopenia, muscle health indicators, diagnostic cutoff, sex-specific reference centiles, age of inflection point

## Abstract

**Background:**

Sarcopenia is an age-related condition, with existing international guidelines predominantly focusing on the elderly and lacking age-specific diagnostic criteria. This study aims to investigate the prevalence of sarcopenia among middle-aged and elderly individuals in the Community, establish reference standards for muscle health indicators stratifiedby gender and age, and analyse age-related patterns of change and the validity of diagnostic cut-offs.

**Methods:**

A cross-sectional design was employed. Between April and June 2025, 2, 631 subjects aged ≥35 years from a community in Shijiazhuang, China, were enrolled and divided into sex-specific subgroups: 35–49, 50–64, and ≥65 years. Muscle Index (SMI), grip strength, and gait speed were measured. Diagnosis was based on the AWGS 2019 criteria. Stratified cutoffs were established using the P20 method, decline inflection points analysed via LOESS, and efficacy validated through ROC curves.

**Results:**

Overall sarcopenia prevalence was 6.65% (8.44% in males, 4.75% in females), rising to 15.26% in the ≥65 age group. Muscle indicators exhibited non-linear age-related decline with sex-specific inflection points; P20 cut-off decreased with age. AUC values for all indicators exceeded 0.75 in both the 50–64 and ≥65 age groups, demonstrating sound diagnostic performance.

**Conclusion:**

This study established sex and age stratified diagnostic cut-offs for sarcopenia in community-dwelling middle-aged and elderly individuals, elucidated the decline characteristics of core muscle indicators, and provided evidence for stratified community screening and precise prevention and control. It aligns with the concept of lifelong muscle health management and offers theoretical support for age-specific interventions.

## 1 Introduction

Sarcopenia is an age-related disease characterized by progressive loss of muscle mass, strength, and physical function. It is closely associated with falls, fractures, disability, and increased all-cause mortality, posing a significant public health challenge in the context of global aging (Cruz-Jentoft et al., 2019; Chen et al., 2020; Chen, 2024; Rosenberg, 1997; Chen et al., 2025a; Asian Working Group for Sarcopenia AWGS, 2025). With the acceleration of global population aging and the trend of sedentary behavior among younger populations, the prevention and control of sarcopenia have expanded to encompass the entire life span. The traditional research focus on the elderly population is no longer sufficient to meet clinical and public health needs 5. Physiologically, skeletal muscle mass begins to decline gradually after age 25, with the rate of decline accelerating significantly after age 50 grip strength declines at a much faster rate than muscle mass, and this process exhibits significant sex and racial differences (Cruz-Jentoft et al., 2019; Chen et al., 2020), further complicating prevention and control strategies. The core logic behind the iterative development of sarcopenia diagnostic criteria lies in the growing emphasis on muscle health across the entire lifespan. Currently, clinical practice still predominantly relies on diagnostic standards based on the elderly population. The 2019 revised version of the European Working Group on Sarcopenia in the Elderly (EWGSOP) guidelines established reduced grip strength as a core indicator, providing a functionally oriented cornerstone for sarcopenia diagnosis (Cruz-Jentoft et al., 2019). The Asian Working Group on Sarcopenia (AWGS) achieved a key breakthrough in its 2025 consensus update by establishing diagnostic reference standards for the 50–64 age group (Chen et al., 2025a). Existing international guidelines do not cover young adults aged 35–49 (Chen et al., 2025a; Baek et al., 2023; Lim et al., 2022; Beaudart et al., 2025). Global urbanization drives lifestyle shifts, making urban populations—characterized by workplace sedentary habits and low exercise participation—high-risk groups for muscle mass and strength decline 5. Physical inactivity, as a core modifiable factor in muscle health decline 10–11, not only accelerates the synergistic decrease in muscle mass and strength 3but is also closely associated with increased risk of adverse metabolic outcomes in midlife 5. This phenomenon has garnered attention in the international sarcopenia field (Cruz-Jentoft et al., 2019; Chen et al., 2025a).

This study aims to establish sex- and age-specific diagnostic cut-offs for sarcopenia in individuals aged ≥35 years. By expanding screening to individuals aged 35 and older, this study identifies the inflection points of muscle decline for different genders, providing clear timelines for early intervention. Additionally, ROC curve analysis validates the diagnostic efficacy of sarcopenia-related health indicators across age groups, offering actionable reference standards for community-based stratified screening. This contributes to the development of targeted prevention and control strategies in clinical and public health practice, advancing muscle health promotion throughout the life span.

## 2 Methods

### 2.1 Study population

This cross-sectional study recruited patients, health examination participants, and accompanying family members visiting the Community Service Center of Lianmeng Subdistrict, Xinhua District, Shijiazhuang City between April 1 and 30 June 2025. Participants were enrolled using consecutive sampling, with inclusion criteria: age ≥35 years, permanent residents residing locally for ≥6 months, voluntary participation, and signed informed consent.

Exclusion criteria: 1. Severe acute or chronic diseases (e.g., active malignancy, end-stage renal disease, decompensated cirrhosis, NYHA Class III-IV heart failure); 2. Major mobility impairments (e.g., amputation, severe arthritis, bedridden status); 3. Major surgery within the past 3 months or current use of medications known to significantly affect muscle metabolism (e.g., systemic corticosteroids, androgen deprivation therapy); 4. Pregnancy or lactation; 5. Inability to undergo reliable assessment due to severe cognitive impairment or psychiatric disorders. A total of 2, 631 subjects were ultimately enrolled, comprising 1, 327 males and 1, 304 females.

### 2.2 Data collection

#### 2.2.1 General data collection

Demographic and anthropometric measurements: sex and age were recorded. Height (accurate to 0.1 cm) and weight (accurate to 0.1 kg) were measured using standard height-weight scales. Participants were instructed to remove shoes and wear light clothing during measurement.

#### 2.2.2 Muscle-related indicators, body fat percentage measurement, and sarcopenia diagnostic criteria (referencing the 2019 Asian Working Group for sarcopenia (AWGS) expert consensus)

##### 2.2.2.1 Core indicator measurements

###### 2.2.2.1.1 Muscle mass measurement

Appendicular skeletal muscle (ASM) in the limbs was measured using a multi-frequency bioelectrical impedance analyzer (Inbody 770, Korea). Standardized conditions were strictly controlled prior to measurement: subjects were required to be in a fasting state or at least 2 h postprandial, had emptied their bladder before measurement, and had not engaged in vigorous exercise within 30 min. During measurement, subjects stood barefoot on the device’s electrode pads, held the electrode handles with both hands, maintained a relaxed posture, and completed the test according to the device manual. SMI was calculated using the formula: SMI (kg/m^2^) = ASM (kg)/height (Chen et al., 2020) (m^2^).

###### 2.2.2.1.2 Grip strength measurement

Grip strength was assessed using a calibrated electronic dynamometer (EH101, Camry, China). During measurement, subjects sat with elbows flexed at 90°, forearms in neutral position, arms hanging naturally while gripping the dynamometer. They applied maximum sustained force, repeating the measurement twice per hand with ≥60 s rest between trials. The highest grip strength value (kg) from either hand was recorded.

###### 2.2.2.1.3 Gait speed measurement

Gait speed was assessed using a 6-m walk test. The testing area consisted of a 10-m straight walkway, with 2 m reserved at each end as acceleration and deceleration zones, and the central 6 m designated as the timed section. Before testing, explain the procedure to the subject, instructing them to walk at their usual pace. Use a stopwatch to record the time taken to traverse the 6-m timed section. Repeat the test twice, and calculate the step speed (m/s) using the faster of the two times. The formula is: Step Speed (m/s) = 6 m/Walking Time (s).

##### 2.2.2.2 Diagnostic criteria for core sarcopenia indicators

Low Muscle Mass: Determined using bioelectrical impedance analysis (BIA), with SMI <7.0 kg/m^2^ for males and SMI <5.7 kg/m^2^ for females; Low grip strength: Reduced grip strength, <28 kg for males, <18 kg for females; Low gait speed: Decreased gait speed, gait speed < 1.0 m/s.

##### 2.2.2.3 Diagnostic criteria for sarcopenia

Sarcopenia is diagnosed when any one of the following conditions is met: Low SMI&low grip strength; Low SMI&low gait speed; Low SMI&low grip strength andlow gait speed.

##### 2.2.2.4 Cutoff point determination

All subjects were stratified by sex and further divided into six age subgroups: 35–49 years, 50–64 years, and ≥65 years. For each sex-age stratum, calculate the 20th percentile (P20) of SMI, grip strength, and gait speed (Chen et al., 2025b), and set these values as the diagnostic cut-offs for low SMI, low grip strength, and low gait speed within that stratum. This P20 method was applied to all sex-age strata in this study.

### 2.3 Statistical analysis

Data processing and analysis were performed using SPSS 26.0 and R 4.5.1 statistical software. Multi-group comparisons were conducted using analysis of variance (ANOVA). For non-normally distributed quantitative data, median (interquartile range) [M (Q1, Q3)] was used for presentation, and Kruskal–Wallis H test was applied for multi-group comparisons. Categorical data were expressed as frequency (n) and percentage (%). Intergroup comparisons were performed using the chi-square test. When chi-square test conditions were not met (any cell with a theoretical frequency <1, or cells with theoretical frequency <5 exceeding 20% of total cells), Fisher’s exact test was used.

For the three core muscle health indicators—SMI, grip strength, and gait speed—percentile methods were used to calculate the fifth percentile (P5), 25th percentile (P25), the 50th percentile (P50), the 75th percentile (P75), and the 95th percentile (P95) for each sex-age stratum, comprehensively presenting the distribution characteristics of these indicators. Simultaneously, the 20th percentile (P20) of these indicators across all sex-age strata was calculated to determine diagnostic cut-offs for low muscle mass, low grip strength, and low physical function. The LOESS model was employed to analyze the continuous age-related trends of SMI, grip strength, and gait speed. Model-fitted curves identified the age-related inflection points for each indicator, visually illustrating the decline patterns of muscle health indicators across sexs. ROC curve analysis evaluated the discriminatory ability of SMI, grip strength, and gait speedfor sarcopenia in the 50–64 age group and ≥65 age group, calculating AUC and its 95% confidence interval. Simultaneously, the diagnostic cutoff points based on the 20th percentile (P20) for each age group were identified on the ROC curves, with corresponding sensitivity and specificity reported to provide a preliminary assessment of the screening performance of the P20 cutoff. AUC values range from 0.5 to 1.0, where AUC values of 0.7–0.8 indicate good diagnostic performance, 0.8–0.9 indicate high diagnostic performance, and ≥0.9 indicate excellent diagnostic performance. All statistical tests were two-sided, with *P* < 0.05 indicating statistically significant differences.

## 3 Results

This study included 2, 631 community residents aged ≥35 years. Through standardized measurements and statistical analysis, we examined the prevalence of sarcopenia, characteristics of core muscle health indicators, and sex-age-specific diagnostic cut-offs. Combined with graphical analysis, the specific results are as follows.

### 3.1 Baseline characteristics and prevalence of sarcopenia

The study population comprised 1, 327 males (50.44%) and 1, 304 females (49.56%). By age group: 857 (32.57%) aged 35–49 years, 758 (28.81%) aged 50–64 years, and 1016 (38.62%) aged ≥65 years.

**FIGURE 1:**
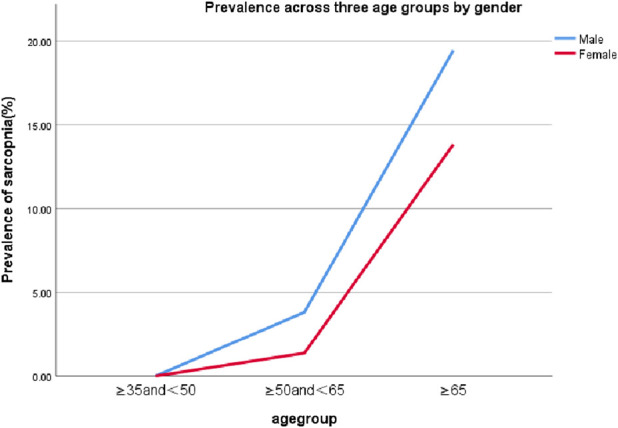
Line graph showing sarcopenia prevalence by sex and age groups.

The overall prevalence of sarcopenia was 6.65% (175/2631), with 8.44% (113/1327) among males and 4.75% (62/1304) among females, showing a statistically significant sex difference (χ^2^ = 14.98, P < 0.05). The sex-age stratified line chart of sarcopenia prevalence demonstrated persistent sex differences and a significant upward trend with increasing age overall (refer Table 1; Figure 1).

**TABLE 1:** Line graph showing sarcopenia prevalence by sex and age groups.

	35–49 years	50–64 years	≥65 years	Χ^2^	P
Male^a^					
Sarcopenia	0	15(3.81%)	98(19.44%)	143.39	<0.05
Non-Sarcopenia	429(100.00%)	379(96.19%)	406(80.56%)		
Female^b^					
Sarcopenia	0	5(1.37%)	57(3.83%)	109.48	<0.05
Non-Sarcopenia	528(100.00%)	359(98.63%)	355(86.17%)		

### 3.2 Distribution characteristics of core muscle health indicators

SMI, grip strength, and gait speed decreased with age across all sexs, with statistically significant differences between age groups (refer Table 2).

**TABLE 2:** Distribution of core muscle health indicators across sex-age groups.

	5th	25th	50th	75th	95th	H	P
Male							
SMI							
35–49 years	7.5	8.1	8.5	8.9	9.8	509.81	<0.05
50–64 years	6.5	7.7	8.0	8.3	8.9		
≥65 years	6.5	7.1	7.4	7.8	8.4		
Grip Strength							
35–49 years	32.0	38.0	44.0	49.3	53.2	621.26	<0.05
50–64 years	25.9	34.4	38.2	44.7	47.0		
≥65 years	24.8	26.4	27.6	33	38.3		
Gait Speed							
35–49 years	1.2	1.3	1.3	1.4	1.5	333.62	<0.05
50-64 years	0.9	1.2	1.3	1.4	1.4		
≥65 years	0.9	1.0	1.1	1.2	1.4		
Female							
SMI							
35–49 years	5.4	6.1	6.5	6.8	7.4	80.74	<0.05
50–64 years	5.4	5.8	6.1	6.7	7.2		
≥65 years	5.0	5.8	6.0	6.5	7.1		
Grip Strength							
35–49 years	21.0	22.0	24.0	28.7	31.0	358.48	<0.05
50–64 years	16.2	20.0	21.0	24.5	29.8		
≥65 years	14.0	18.1	19.7	22.3	27.6		
Gait Speed							
35–49 years	1.2	1.3	1.4	1.5	1.5	584.31	<0.05
50–64 years	0.9	1.2	1.2	1.3	1.5		
≥65 years	0.9	1.0	1.1	1.2	1.4		

Post-hoc pairwise comparisons were further performed for each indicator using Dunn’s test with Bonferroni correction,and the results showed that all pairwise comparisons of each indicator among different age groups were statistically significant (all *P* < 0.05).

### 3.3 Age-sex-specific diagnostic cutoffs (P20)

Diagnostic cut-offs for low SMI, low grip strength, and low geit speed were determined using the percentile method for each sex-age stratum (refer Table 3):

**TABLE 3:** Diagnostic cutoffs (P20) of muscle health-related indicators across different sex-age groups.

​	Observations	SMI	Grip strength	Gait speed
Male				
35–49 years	429	8.0	38.0	1.3
50–64 years	394	7.7	34.0	1.2
≥65 years	504	7.0	26.2	1.0
Female				
35–49 years	528	6.0	22.0	1.3
50–64 years	364	5.7	20.0	1.2
≥65 years	412	5.7	18.0	1.0

Males: SMI decreased from 8.0 kg/m^2^ (ages 35–49) to 7.0 kg/m^2^ (≥65 years); grip strength decreased from 38.0 kg (ages 35–49) to 26.2 kg (≥65 years); gait speeddecreased from 1.3 m/s for ages 35–49 to 1.0 m/s for ages ≥65. Females: SMI was 6.0 kg/m^2^ for ages 35–49, 50–64 years and ≥65 years both 5.7 kg/m^2^; grip strength decreased from 22.0 kg in 35–49 years to 18.0 kg in ≥65 years; gait speed decreased from 1.3 m/s in 35–49 years to 1.0 m/s in ≥65 years.

### 3.4 LOESS curve-smoothing visualization results

**FIGURE 2:**
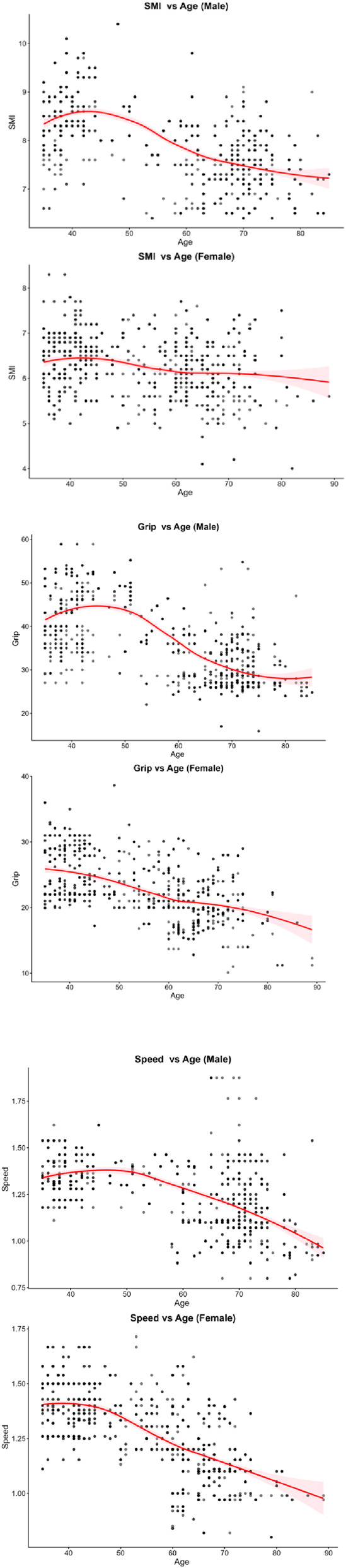
The non-linear relationship between age at SMI, grip and gait speed by sex.

Using age as the independent variable and SMI, grip strength, and gait speed as dependent variables, scatter plots were plotted. Subsequently, the LOESS method was applied to fit age-related trends for SMI, grip strength, and gait speed across sexs. Results indicate that SMI, grip strength, and gait speed generally decline with increasing age. The rate of decline for grip strength exceeds that of SMI and gait speed. Men exhibit faster rates of decline in SMI and grip strength than women, with the onset of decline occurring earlier in men (around age 43) than in women (around age 46). The inflection point for gait speed decline occurs between ages 46–47 in men and 39–40 in women (refer Figure 2).

### 3.5 ROC validation of P20 diagnostic cutoff for muscle health indicators

**FIGURE 3:**
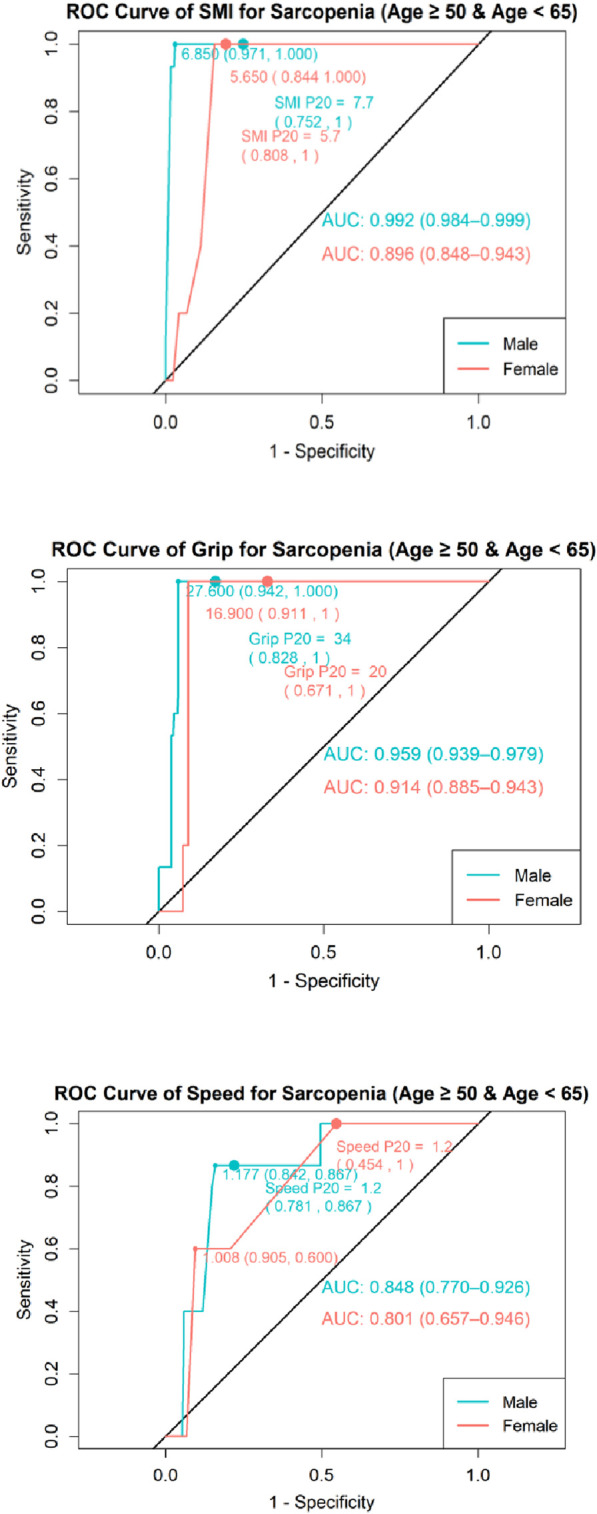
ROC curves for validating P20 cutoffs of SMI, grip strength, and gait speedin sarcopenia diagnosis (Aged 50–64 years).

**FIGURE 4:**
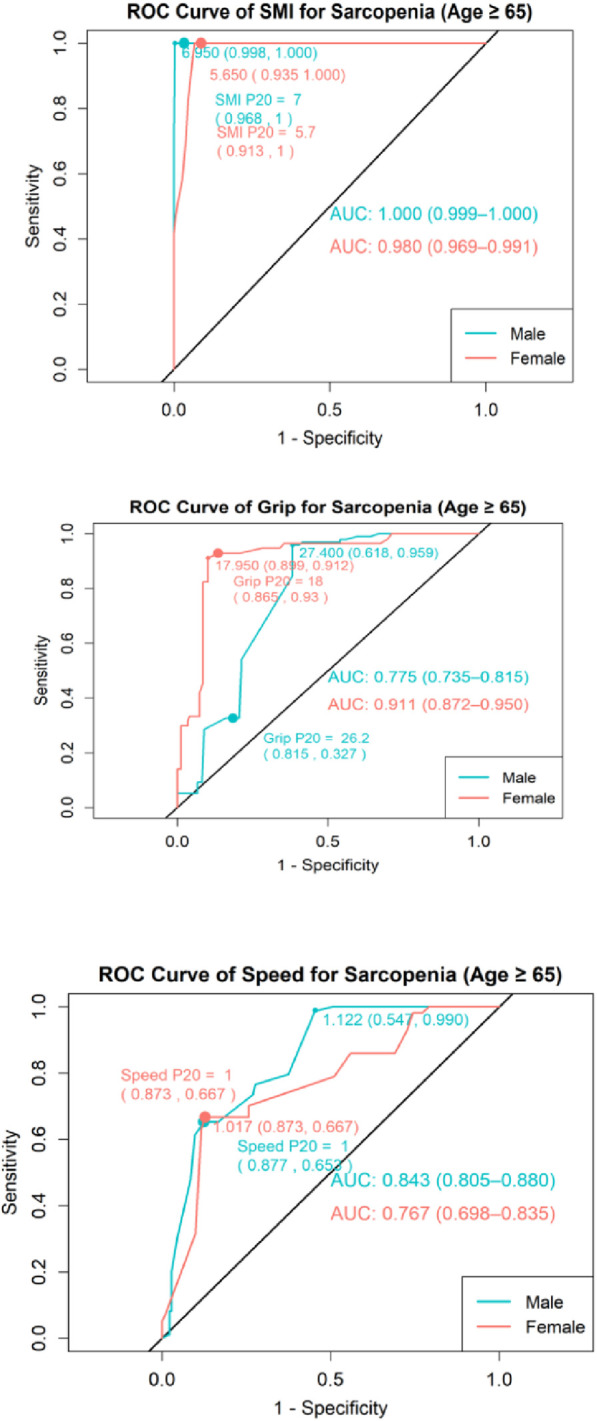
ROC curves for validating P20 cutoffs of SMI, grip strength, and gait speedin sarcopenia diagnosis (aged ≥ 65 years).

ROC curve analysis evaluated the discriminatory ability of SMI, grip strength, and gait speed for sarcopenia in the 50–64 age group and ≥65 age group. Results showed that the AUC for all indicators exceeded 0.75, indicating that the P20 diagnostic cutoff demonstrated good diagnostic efficacy (refer Figures 3, 4).

## 4 Discussion

Based on cross-sectional data from 2, 631 community-dwelling middle-aged and elderly individuals, this study systematically elucidated the epidemiological characteristics of sarcopenia and the sex-age-specific decline patterns of core muscle health indicators. It established and validated a sex- and age-stratified diagnostic cutoff system applicable to individuals aged ≥35 years.

The overall prevalence of sarcopenia in individuals aged ≥35 years was 6.65%, with males (8.44%) exhibiting higher rates than females (4.75%). Prevalence significantly increased with age, reaching 15.26% in the ≥65-year-old group. This finding aligns with analyses by Weng, S. E. Mayhew AJ, et al. (Weng et al., 2025; Mayhew et al., 2019), where the prevalence of sarcopenia in Asian populations aged ≥60 years ranged from 13.2% to 19.9%. This confirms the age-related epidemiological characteristics of sarcopenia in Asian populations. In this study, the middle-aged cohort aged 50–64 already showed clear signs of sarcopenia onset (3.81% in men, 1.37% in women). This finding aligns closely with the conclusions of the Asian Working Group on Sarcopenia (AWGS) 2025 Consensus 5and the findings of multinational cohort studies by Chen LK et al. (Chen et al., 2025b). The AWGS 2025 Consensus breaks from the previous focus on individuals aged 65 and older, for the first time incorporating the 50–64 age group into screening and intervention frameworks. The core rationale is that muscle health decline in midlife represents a pre-symptomatic phase for adverse outcomes in older age, such as sarcopenia and falls. Although sub clinical muscle mass loss and progressive declines in grip strength and physical function during this stage do not meet geriatric diagnostic criteria, they possess potential for reversible intervention. Analysis by Chen LK et al.12 of subjects from eight cohort studies in Japan, Malaysia, and Taiwan further confirms that muscle health decline in Asian populations exhibits a middle-age onset pattern, suggesting this stage reflects the combined effects of age-related physiological decline and lifestyle factors. Without intervention, sub clinical muscle function decline after age 35 becomes a major precursor to high sarcopenia incidence after age 50 (Beaudart et al., 2025; Chen et al., 2025b; Petermann-Rocha et al., 2022; Auyeung et al., 2020).

LOESS regression analysis revealed nonlinear decline patterns in muscle health indicators with significant sex differences. This aligns with findings by Good paster, Zhang, S, et al. (Goodpaster et al., 2006; Zhang et al., 2024; Petermann-Rocha et al., 2022; Shafiee et al., 2017) that grip strength declines at a much faster rate than muscle mass, while further detailing sex-specific differences in decline inflection points and rates:

Regarding SMI decline, the inflection point occurs earlier in men (44–46 years) than in women (around 50 years), while SMI stabilizes in women after age 50 (with breakpoints of 5.7 kg/m^2^ for both the 50–64 age group and ≥65 age group). This sex difference may be closely linked to hormonal changes: after age 40, men experience a gradual annual decline in androgen levels, which are key regulators of myosin synthesis and inhibitors of muscle cell apoptosis 20; in women, the protective effect of estrogen on muscle tissue delays muscle mass decline until post menopause, with limited further decline thereafter (Chen et al., 2020; Chen, 2024; Rosenberg, 1997; Chen et al., 2025a). Furthermore, the age stability of SMI in women observed in this study aligns with the muscle mass distribution characteristics reported for Asian women in the AWGS 2025 consensus (Chen et al., 2025a), reflecting the racial specificity of muscle aging trajectories. Regarding grip strength decline, both sexes begin to decline around age 40.

The grip strength cutoff point for men aged ≥65 (26.2 kg) approaches the low grip strength diagnostic threshold (28 kg) recommended by the AWGS 2019 guidelines, while the cutoff point for women aged ≥65 (18.0 kg) aligns with the guideline threshold (18 kg). This disparity likely stems from innate differences in muscle fiber composition between gender, with men possessing a higher proportion of fast-twitch fibers. Fast-twitch fibers exhibit greater sensitivity to age-related neuro muscular functional decline (Riviati and Indra, 2023). Concurrently, factors such as reduced occupational labor intensity and decreased physical activity participation during middle age further accelerate grip strength loss in men (Chen et al., 2025a).

Regarding gait speed decline, gender differences show women initiating decline earlier while men accelerate later, the inflection point for women occurs at age 45, compared to age 54 for men. However, the magnitude of gait speed decline in older men (32.4% reduction from peak) is slightly greater than that in women (30.3% reduction). This aligns with the systematic review findings by Riviati et al. (Riviati and Indra, 2023), suggesting that muscle function impairment may precede significant muscle mass decline. The relatively low exercise participation rates among women during middle age and the differential impact of repetitive household tasks on physical function 20 may be key factors contributing to their earlier gait speed decline. Furthermore, as a comprehensive indicator of physical function, the sex-specific decline pattern of gait speed suggests that women should focus on maintaining physical function earlier, while men should prioritize preventing falls associated with rapid gait speed decline in old age. Regarding decline rates, middle-aged men (45–65 years) exhibited significantly higher rates of SMI and grip strength decline than women, with sustained high rates of loss persisting into old age. In contrast, women showed markedly slower loss rates in old age, potentially due to lower baseline muscle mass, limited scope for further decline in old age, and residual muscle-protective effects of estrogen (Kaspy et al., 2024; li et al., 2024; Gao et al., 2021; Lee et al., 2023; Izquierdo et al., 2025). As a core indicator of physical function, the early decline in gait speed among women in middle age suggests that muscle function impairment may precede significant muscle mass loss. This finding aligns closely with the assessment principle proposed by the GLIS Working Group 9 that functional indicators should take precedence over structural indicators.

This study employed a percentile-based approach (P20) to establish sex- and age-specific diagnostic thresholds covering 35–49 years, 50–64 years, and ≥65 years. This methodology adheres to the percentile-based principle recommended by the AWGS 2025 Consensus5while employing a stratified design to accommodate physiological characteristics across age groups. This approach avoids over diagnosis in younger adults or under diagnosis in the elderly that a single cutoff might cause. It reflects age-related natural decline while ensuring diagnostic specificity across age groups, consistent with the trend in standardized grip strength data for Asian populations reported by Auyeung et al. (Auyeung et al., 2020), further validating the scientific basis of the cutoffs.

This study makes several significant contributions: Firstly, with a substantial sample size (2, 631 participants) covering all age groups—35–49, 50–64, and ≥65 years—and a balanced male-female ratio, it accurately reflects the prevalence characteristics of sarcopenia among middle-aged and elderly individuals in the community, significantly enhancing the study’s external validity. Secondly, it overcomes the traditional limitation of focusing solely on the elderly by extending screening to individuals aged 35 and above for the first time. It systematically assesses the age- and sex-specific decline patterns of three core indicators—SMI, grip strength, and gait speed, identifying the inflection points for decline in each metric, thereby deepening our understanding of the mechanisms underlying muscle health deterioration. Thirdly, a stratified diagnostic cutoff system was established using the percentile method (P20). ROC curve validation confirmed its robust diagnostic efficacy (AUC consistently >0.75), coupled with operational simplicity and suitability for community clinical settings. This provides a directly applicable tool for stratified screening and precision prevention of sarcopenia. Overall, this study aligns with the concept of lifelong muscle health management, providing crucial evidence for developing differentiated intervention strategies. It also lays the groundwork for future longitudinal studies to validate the stability of inflection points and multi centre research to expand its application.

Several limitations of this study should be acknowledged. The cross-sectional design cannot establish a causal relationship between sarcopenia and risk factors, nor capture long-term trajectories of muscle health. Future longitudinal studies are needed to validate the stability of the inflection point. The sample originates from a single urban community, introducing geographical selection bias. Extrapolating results to other regions requires caution; multicenter studies will be conducted subsequently. Muscle mass was measured using BIA, which is suitable for community clinical settings but less accurate than the DXA gold standard. DXA will be used in future studies to further validate the cutoff points. Confounding factors such as exercise frequency, dietary nutrition, and hormone levels were not included, preventing a comprehensive analysis of their modulatory effects on muscle health decline.

## 5 Conclusion

In summary, this study systematically elucidates the sex- and age-specific decline patterns of muscle health indicators (SMI, grip strength, gait speed) in community-dwelling older adults. It identifies the inflection points for decline in each indicator and establishes and validates diagnostic reference standards for each. This framework adheres to the AWGS 2025 consensus recommendations while adapting to the physiological decline characteristics of different age groups. It effectively prevents overdiagnosis or missed diagnosis, supports the development of differentiated clinical intervention strategies, and ultimately reduces the prevalence of sarcopenia in older adults and the risk of related adverse outcomes (falls, fractures, disability). This provides a new prevention and control approach to alleviate the health burden of aging.

## Data Availability

The original contributions presented in the study are included in the article/supplementary material, further inquiries can be directed to the corresponding author.

## References

[B1] Asian Working Group for Sarcopenia (AWGS) (2025). Revised Asian consensus on sarcopenia diagnosis and management 2025. J. Cachexia, Sarcopenia Muscle 16 (11), 2897–2912.

[B2] Auyeung T. W. Arai H. Chen L. K. Woo J. (2020). Letter to the editor: normative data of handgrip strength in 26344 older adults - a pooled dataset from eight cohorts in Asia. J. Nutr. Health Aging. 24 (1), 125–126. 10.1007/s12603-019-1287-6 31886819 PMC12879206

[B3] Baek J. Y. Jung H. W. Kim K. M. Kim M. Park C. Y. Lee K. P. (2023). Korean Working Group on Sarcopenia Guideline: expert consensus on sarcopenia screening and diagnosis by the Korean Society of sarcopenia, the Korean Society for Bone and Mineral research, and the Korean Geriatrics Society. Ann. Geriatr. Med. Res. 27, 9–21. 10.4235/agmr.23.0009 36958807 PMC10073972

[B4] Beaudart C. Alcazar J. Aprahamian I. Batsis J. A. Yamada Y. Prado C. M. (2025). Health outcomes of sarcopenia: a consensus report by the outcome working group of the Global Leadership Initiative in sarcopenia (GLIS). Aging Clin. Exp. Res. 37 (1), 100. 10.1007/s40520-025-02995-9 40120052 PMC11929733

[B5] Chen L. K. (2024). Sarcopenia in the era of precision health: toward personalized interventions for healthy longevity. J. Chin. Med. Assoc. 87 (11), 980–987. 10.1097/JCMA.0000000000001164 39257038 PMC12718906

[B6] Chen L. K. Woo J. Assantachai P. Auyeung T. W. Chou M. Y. Iijima K. (2020). Asian Working Group for Sarcopenia: 2019 consensus update on sarcopenia diagnosis and treatment. J. Am. Med. Dir. Assoc. 21 (3), 300–307.e2. 10.1016/j.jamda.2019.12.012 32033882

[B7] Chen L. K. Hsiao F. Y. Akishita M. Assantachai P. Lee W. J. Lim W. S. (2025a). A focus shift from sarcopenia to muscle health in the Asian Working Group for Sarcopenia 2025 Consensus Update. J. Nat. Aging 5 (11), 2164–2175. 10.1038/s43587-025-01004-y 41188603

[B8] Chen L. K. Meng L. C. Peng L. N. Lee W. J. Zhang S. Nishita Y. (2025b). Mapping normative muscle health metrics across the aging continuum: a multinational Study pooling data from eight cohorts in Japan, Malaysia and Taiwan. J. Cachexia Sarcopenia Muscle 16 (1), e13731. 10.1002/jcsm.13731 39971708 PMC11839280

[B9] Cruz-Jentoft A. J. Bahat G. Bauer J. Boirie Y. Bruyère O. Cederholm T. (2019). Sarcopenia: revised European consensus on definition and diagnosis. J. Age Ageing 48 (1), 16–31. 10.1093/ageing/afy169 30312372 PMC6322506

[B17] Gao Q. Hu K. Yan C. Zhao B. Mei F. Chen F. (2021). Associated factors of sarcopenia in community-dwelling older adults: a systematic review and meta-analysis. J. Nutr. 13 (12), 4291. 10.3390/nu13124291 34959843 PMC8707132

[B10] Goodpaster B. H. Park S. W. Harris T. B. Kritchevsky S. B. Nevitt M. Schwartz A. V. (2006). The loss of skeletal grip strength, mass, and quality in older adults: the health, aging and body composition study. j. Gerontology Ser. A Biol. Sci. Med. Sci. 61 (10), 1059–1064. 10.1093/gerona/61.10.1059 17077199

[B11] Izquierdo M. Barreto P. S. Arai H. Bischoff-Ferrari H. A. Cadore E. L. Cesari M. (2025). Global consensus on optimal exercise recommendations for enhancing healthy longevity in older adults (ICFSR). J. Nutr. Health Aging 29 (1), 100401. 10.1016/j.jnha.2024.100401 39743381 PMC11812118

[B12] Kaspy M. S. Hannaian S. J. Bell Z. W. Churchward-Venne T. A. (2024). The effects of branched-chain amino acids on muscle protein synthesis, muscle protein breakdown and associated molecular signalling responses in humans: an update. J.Nutr Res. Rev. 37 (2), 273–286. 10.1017/S0954422423000197 37681443

[B13] Lee W. J. Peng L. N. Lin M. H. Loh C. H. Chen L. K. (2023). Letter to the editor: disentangling mortality associations: an in-depth comparative study of possible sarcopenia *versus* sarcopenia of AWGS 2019. J. Nutr. Health Aging 27, 685–686. 10.1007/s12603-023-1953-6 37702345 PMC12877654

[B18] Li Q. Cheng H. Cen W. Yang T. Tao S. (2024). Affiliations expand development and validation of a predictive model for the risk of sarcopenia in the older adults in China. J. Eur J. Med. Res. 29 (1), 278. 10.1186/s40001-024-01873-w 38725036 PMC11084063

[B14] Lim W. S. Cheong C. Y. Lim J. P. Tan M. M. Y. Chia J. Q. Malik N. A. (2022). Singapore clinical practice guidelines for sarcopenia: screening, diagnosis, management and prevention. J. Frailty Aging 11 (4), 348–369. 10.14283/jfa.2022.59 36346721

[B15] Mayhew A. J. Amog K. Phillips S. Parise G. McNicholas P. D. de Souza R. J. (2019). The prevalence of sarcopenia in community-dwelling older adults, an exploration of differences between studies and within definitions: a systematic review and meta-analyses. Age Ageing 48 (1), 48–56. 10.1093/ageing/afy106 30052707

[B16] Petermann-Rocha F. Balntzi V. Gray S. R. Lara J. Ho F. K. Pell J. P. (2022). Global prevalence of sarcopenia and severe sarcopenia: a systematic review and meta-analysis. J. Cachexia Sarcopenia Muscle 13 (1), 86–99. 10.1002/jcsm.12783 34816624 PMC8818604

[B19] Riviati N. Indra B. (2023). Relationship between muscle mass and grip strength with physical performance in older adults: a systematic review. SAGE Open Med. 27, 11. 10.1177/20503121231214650 PMC10683395 38033420

[B20] Rosenberg I. H. (1997). Sarcopenia: origins and clinical relevance. J. Nutr. 127 (5 Suppl. l), 990S–991S. 10.1093/jn/127.5.990S 9164280

[B21] Shafiee G. Keshtkar A. Soltani A. Ahadi Z. Larijani B. Heshmat R. (2017). Prevalence of sarcopenia in the world: a systematic review and meta-analysis of general population studies. J. Diabetes Metab. Disord. 16, 10.1186/s40200-017-0302-x 28523252 PMC5434551

[B22] Weng S. E. Huang Y. W. Tseng Y. C. Peng H. R. Lai H. Y. Akishita M. (2025). The evolving landscape of sarcopenia in Asia: a systematic review and meta-analysis following the 2019 Asian working group for sarcopenia (AWGS) diagnostic criteria. Arch. Gerontol. Geriatr. 128, 105596. 10.1016/j.archger.2024.105596 39232423

[B23] Zhang S. Peng L. N. Lee W. J. Nishita Y. Otsuka R. Arai H. (2024). Muscle function outweighs appendicular lean mass in predicting adverse outcomes: evidence from Asian longitudinal studies. J. Nutr. Health Aging. 28 (12), 100403. 10.1016/j.jnha.2024.100403 39476465 PMC12877261

